# Transmission of the eyeworm *Thelazia callipaeda*: between fantasy and reality

**DOI:** 10.1186/s13071-015-0881-7

**Published:** 2015-05-14

**Authors:** Domenico Otranto, Filipe Dantas-Torres

**Affiliations:** Department of Veterinary Medicine, Università degli Studi di Bari, Valenzano, Bari, Italy; Department of Immunology, Centro de Pesquisas Aggeu Magalhães, Fundação Oswaldo Cruz, 50740465 Recife, Pernambuco Brazil

**Keywords:** *Thelazia callipaeda*, Human infection, Vector, *Phortica varigata*, Ophthalmology

## Abstract

**Background:**

*Thelazia callipaeda* is transmitted by *Phortica variegata*, a drosophilid that feeds on lachrymal secretions of mammals. Scientific information on human thelaziosis is still relatively limited, mainly for physicians and ophthalmologists. Indeed, the literature is full of misleading information on the transmission of *T. callipaeda* to humans.

**Findings:**

A recent paper reported a case of human intraocular infestation in a patient from Karnataka. The information presented in that article as well as in other articles in the international literature is outdated and incorrect in several instances, mostly regarding to the localization of *T. callipaeda* in the host, its biology and routes of transmission.

**Conclusions:**

Physicians and ophthalmologists should be aware that *T. callipaeda* is larviparous and transmitted exclusively by secretophagous flies. These flies buzz around the eyes of animals and humans at the daytime, landing on the eyes and releasing the infective larvae on the host conjunctiva. That is the only possible way of transmission of *T. callipaeda*.

## Findings

**Fig. 1:**
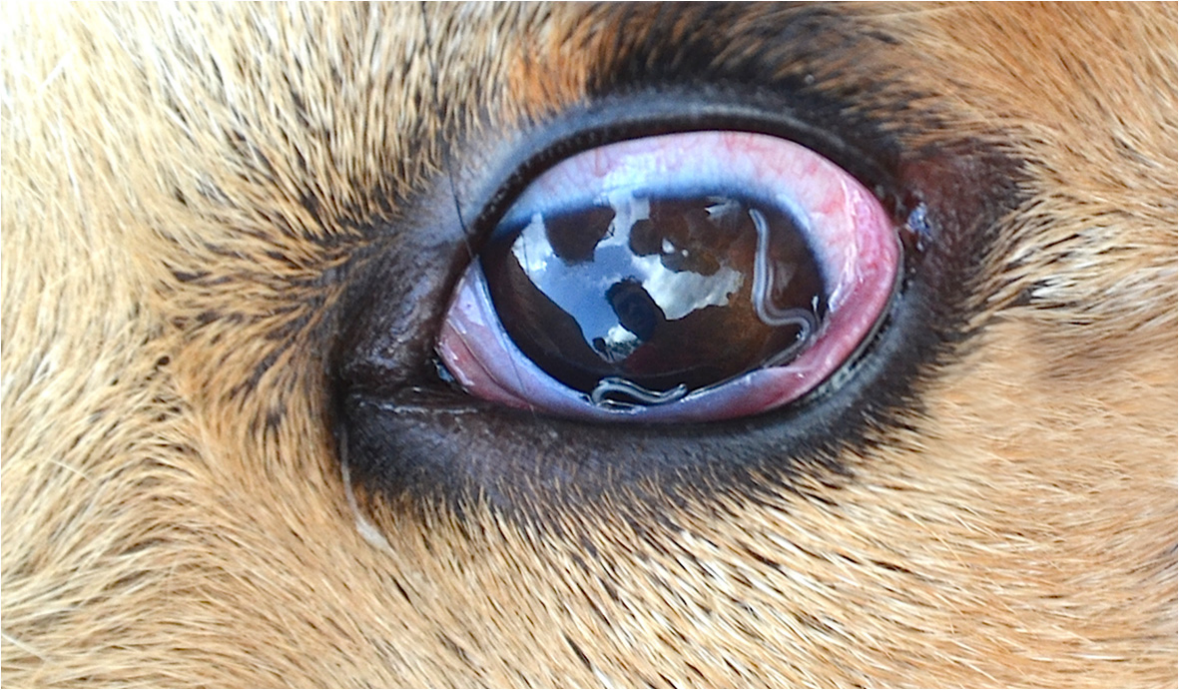
Dog eye with *Thelazia callipaeda* worms. Nematodes in the conjunctiva of a dog with a severe conjunctivitis and follicular hypertrophy of the conjunctiva

*Thelazia callipaeda* (Spirurida, Thelazidae) has been known for a long time as the “oriental eye-worm” due to its geographical distribution in the former Soviet Republics and in many far eastern countries. However, it is now evident that *T. callipaeda* is not confined in far Eastern Asian countries (e.g., India, Thailand, China and Japan). Indeed, it has been increasingly reported in dogs, cats and wild carnivores from Europe [1]. Importantly, where the infection is well established in domestic (dogs and cats) and wild carnivores such as foxes, beech martens and wild cats [2], cases of human thelaziosis may occur (e.g., France, Italy and Spain) [3, 4], indicating a relationship between the infection in humans and other susceptible animals. The strict relationship between the biological life cycle of this parasite in humans and other animals (Fig. 1) is also demonstrated by the occurrence of a single haplotype (i.e., h1) of *T. callipaeda* among different host species in Europe [5]. Foxes play a central role as wild hosts of the infection as indicated by the high prevalence of thelaziosis recorded in this animal species in hyperendemic areas of southern Italy (49.3 %) [2]. Since the first report in Italy [6], the infection by *T. callipaeda* has been recorded in many European countries including France [7], Switzerland [8], Spain [9], Portugal [10] and even eastern Europe [11, 12]. The growing awareness of parasitologists and veterinary practitioners has most likely contributed to the increased number of reports of thelaziosis by *T. callipaeda* in Europe. However, the rather apparent clinical presentation (mild conjunctivitis, follicular hypertrophy of the conjunctiva, foreign body sensation, epiphora, itchiness, congestion, swelling, hypersensitivity to light, and keratitis) may have rendered the diagnosis of thelaziosis not so difficult, suggesting the recent introduction of *T. callipaeda* in some areas.

The high number of reports of eyeworm infestation throughout the last few years parallels the scientific information recently acquired on the biology of this nematode in both the definitive and intermediate hosts. Indeed, *T. callipaeda* is transmitted by *Phortica variegata*, a drosophilid that feeds on lachrymal secretions of mammals [13, 14]. Importantly, for this parasite, information gained in veterinary parasitology is as yet more refined than in human medicine, therefore emphasizing the importance of the One Health approach in medical sciences. For example, it is well known that, in southern Italy, *P. variegata* has a seasonal pattern (from March to October) and a predominately crepuscular activity that overlaps the behaviour of wild hosts [15].

Although the high prevalence of eyeworms in dogs and wildlife represents an alert for human populations, scientific information on human thelaziosis is still limited, mainly for physicians and ophthalmologists. Indeed, the literature is full of misleading information on the transmission of *T. callipaeda* to humans. For example, a recent paper attracted our attention due to its imaginative nature and misleading conclusions. The article entitled “Human ocular thelaziasis in Karnataka” [16], recently published in the open access Indian Journal of Ophthalmology, reported a case of human intraocular infestation in a patient from Karnataka. The authors attempted to put the clinical case under a broad context, describing the main epidemiological and biological features of human thelaziosis. Regrettably, the information presented in that article is outdated and incorrect in several instances. For example, China (and not India) is the country with the highest number of cases of human thelaziosis [17]. Although the infection is mostly linked to the rural areas where the insect vector perpetuates, it has never been demonstrated that “Cattle rearing, contact with stray dogs […] make the humans vulnerable to ocular *Thelaziasis*.” In addition, *T. callipaeda* localizes on the conjunctiva under the eyelids and not inside the eye of the patients, therefore making rather awkward the meaning of the reasons why “conjunctival and cornel injuries, traumatic conjunctivitis facilitate the introduction of the larvae into the sub-conjunctival space and vitreous cavity”.

Importantly, in spite of the large amount of data available in the international literature [13, 14] basic information on the life history of this parasite is completely ignored by the authors, who used inappropriate arguments in their attempt to explain the potential transmission routes for this parasite. The authors stated “the main mode of transmission in this case was probably injury with the cattle tail and might be through contaminated towels”. Third-stage infective larvae of *T. callipaeda* are only transmitted by secretophagous flies feeding on the human eyes; other ways of transmission for these larvae is not possible. In the past, other authors have hypothesized other fanciful ways of transmission. For example, some authors reported that the life cycle of *T. callipaeda* remains unclear and discussed the possibility of human infection through the skin or by drinking untreated water [18]. Nonetheless the level of imagination reached in the abovementioned article has never found equals in that authors stated that the infection occurred with “the towels being contaminated with cow dung containing deposited eggs/larvae of the worm and the same being used for wiping or rubbing the eyes after the injury as the possible mode of entry of eggs/larvae into the eyes”.

## Conclusions

Physicians and ophthalmologists with no experience in parasitology should be aware that *T. callipaeda* is larviparous and transmitted exclusively by secretophagous flies. These flies buzz around the eyes of animals and humans at the daytime, finally landing on the eyes and releasing the infective larvae on the host conjunctiva. Therefore, the prevention of thelaziasis is difficult to manage using protective bed nets at night, as suggested by Krishnachary and colleagues [16], unless the authors intended to prevent mosquito-transmitted diseases, such as malaria.
